# Global Profiling of Rice and Poplar Transcriptomes Highlights Key Conserved Circadian-Controlled Pathways and *cis*-Regulatory Modules

**DOI:** 10.1371/journal.pone.0016907

**Published:** 2011-06-09

**Authors:** Sergei A. Filichkin, Ghislain Breton, Henry D. Priest, Palitha Dharmawardhana, Pankaj Jaiswal, Samuel E. Fox, Todd P. Michael, Joanne Chory, Steve A. Kay, Todd C. Mockler

**Affiliations:** 1 Department of Botany and Plant Pathology, Center for Genome Research and Biocomputing, Oregon State University, Corvallis, Oregon, United States of America; 2 Section of Cell and Developmental Biology, University of California San Diego, La Jolla, California, United States of America; 3 The Waksman Institute of Microbiology, Rutgers, The State University of New Jersey, Piscataway, New Jersey, United States of America; 4 Plant Biology Laboratory, The Salk Institute for Biological Studies and Howard Hughes Medical Institute, La Jolla, California, United States of America; Instituto de Biología Molecular y Celular de Plantas, Spain

## Abstract

**Background:**

Circadian clocks provide an adaptive advantage through anticipation of daily and seasonal environmental changes. In plants, the central clock oscillator is regulated by several interlocking feedback loops. It was shown that a substantial proportion of the Arabidopsis genome cycles with phases of peak expression covering the entire day. Synchronized transcriptome cycling is driven through an extensive network of diurnal and clock-regulated transcription factors and their target *cis*-regulatory elements. Study of the cycling transcriptome in other plant species could thus help elucidate the similarities and differences and identify hubs of regulation common to monocot and dicot plants.

**Methodology/Principal Findings:**

Using a combination of oligonucleotide microarrays and data mining pipelines, we examined daily rhythms in gene expression in one monocotyledonous and one dicotyledonous plant, rice and poplar, respectively. Cycling transcriptomes were interrogated under different diurnal (driven) and circadian (free running) light and temperature conditions. Collectively, photocycles and thermocycles regulated about 60% of the expressed nuclear genes in rice and poplar. Depending on the condition tested, up to one third of oscillating Arabidopsis-poplar-rice orthologs were phased within three hours of each other suggesting a high degree of conservation in terms of rhythmic gene expression. We identified clusters of rhythmically co-expressed genes and searched their promoter sequences to identify phase-specific *cis*-elements, including elements that were conserved in the promoters of Arabidopsis, poplar, and rice.

**Conclusions/Significance:**

Our results show that the cycling patterns of many circadian clock genes are highly conserved across poplar, rice, and Arabidopsis. The expression of many orthologous genes in key metabolic and regulatory pathways is diurnal and/or circadian regulated and phased to similar times of day. Our results confirm previous findings in Arabidopsis of three major classes of *cis*-regulatory modules within the plant circadian network: the morning (ME, GBOX), evening (EE, GATA), and midnight (PBX/TBX/SBX) modules. Identification of identical overrepresented motifs in the promoters of cycling genes from different species suggests that the core diurnal/circadian *cis*-regulatory network is deeply conserved between mono- and dicotyledonous species.

## Introduction

Organisms living at the surface of the Earth evolved circadian clocks to synchronize internal biology with the external environmental cycles of light and temperature. Plants experience daily changes in photo- and thermocycles that encode valuable information in regards to time of day and season. The circadian clock is coupled to gene expression and helps coordinate physiological and metabolic processes with daily and seasonal environmental changes. This coordination results in increased fitness, growth vigor and enhanced adaptation to the changing environment [1].

Prior studies in Arabidopsis suggested that a large proportion of the transcriptome was subjected to diurnal/circadian regulation. Early microarray time course data in Arabidopsis estimated that between 6% and 15% of the Arabidopsis transcriptome is regulated by the circadian clock [2], [3], [4]. In comparison, an enhancer trap study predicted that the circadian clock regulates about 36% of Arabidopsis genes [5]. Looking at light/dark-entrained seedlings, Blasing et al. [6] estimated that 30–50% of the Arabidopsis transcriptome cycled under photocycles and continuous temperature. More recent microarray experiments involving an extensive panel of diurnal (light, temperature cycles) and free running (circadian) conditions showed that rhythmically expressed Arabidopsis genes cover all times of day [1], with collectively, up to 89% of detectable Arabidopsis transcripts cycling under at least one diurnal or circadian condition. Whether or not a similar pattern is present in other higher plants is still an unanswered question.

The plant core clock is regulated by several interconnected negative and positive feedback loops resulting in circadian rhythms with a period of about twenty-four hours (reviewed in [7]). Environmental signals such as light and temperature are integrated by a clock input while the output circuits modulate multiple biochemical and regulatory pathways. The core oscillator feedback loop is regulated by the two morning-expressed transcription factors: CIRCADIAN CLOCK ASSOCIATED 1 (CCA1) and LATE ELONGATED HYPOCOTYL (LHY) [8], [9], [10] and by the evening-expressed TIMING OF CAB EXPRESSION 1 (TOC1). In line with this model, the oscillating patterns of most Arabidopsis cycling genes peak near dusk and dawn [1], [11].

In this study we interrogated the transcriptomes of the monocotyledonous plant rice, *Oryza sativa*, *ssp. japonica* [12] and a dicotyledonous plant poplar, *Populus trichocarpa*, clone *Nisqually1* [13] using a genome-scale microarray time course approach. A sizable proportion of rice and poplar genes displayed daily oscillations in transcript abundance under different diurnal and circadian conditions. Similar to Arabidopsis, the rhythmic expression of rice and poplar transcripts encompassed all hours of the day, with most genes peaking just before dawn or dusk. Conspicuously, a large proportion of the transcripts encoding putative transcription factors in both species also showed regulation by light and temperature. We identified clusters of genes co-expressed at specific times of day and searched their promoter sequences for overrepresented potential *cis*-regulatory elements. Comparisons of the distributions of phase-specific *cis*-elements in the promoters of co-expressed genes in Arabidopsis, rice, and poplar genomes revealed numerous circadian-associated promoter elements. The conservation of the circadian clock across plant phyla coupled with the resolution of the diurnal/circadian time courses enabled genome-scale identification of co-regulated genes and presents a robust system to study the evolution of transcriptional networks.

## Results

### Photocycles and thermocycles drive diurnal gene expression in poplar and rice

To enable side-by-side comparisons between poplar and rice, we employed similar diurnal and circadian time course schemes (under the respective species-specific physiological ranges of temperature and light intensity) (see **Figure S1** and Materials and Methods). Three time courses were performed in diurnal (driven) conditions for each species: photocycles alone (12 hrs light/12 hrs dark; 12 hrs hot/12 hrs hot: LDHH), photo/thermocycles together (12 hrs light/12 hrs dark; 12 hrs hot/12 hrs cold: LDHC), and thermocycles (12 hrs light/12 hrs light; 12 hrs hot/12 hrs cold: LLHC). To capture the circadian-regulated transcriptome oscillations a circadian (free running; LL continuous 24 hrs of light) segment under continuous light followed each diurnal time course. Total RNA was sampled every four hours during both driven and free running segments of each time course. To ensure a complete transition from driven to free running conditions and to identify transcripts that cycle robustly in both diurnal and circadian segments, plants were exposed to continuous light for forty eight hours prior to sampling for the circadian time course.

To interrogate daily changes in transcript abundance in rice and poplar, we adapted the computational pipeline previously developed for Arabidopsis [1], [11]. Normalized genome-scale microarray time course datasets (ArrayExpress accession numbers E-MEXP-2506 and E-MEXP-2509) were processed using the model-based pattern-matching algorithm HAYSTACK ([1], [11], http://haystack.cgrb.oregonstate.edu/). A series of statistical tests were used to identify the best-fit model, phase-of-expression, *p*-value, and false discovery rate (FDR) for each gene. Identification of oscillating transcripts was done using the best matching HAYSTACK model with a correlation cutoff value of ≥0.75 corresponding to a ∼5% FDR (derived by permutation analysis; [1]).

**Figure 1:**
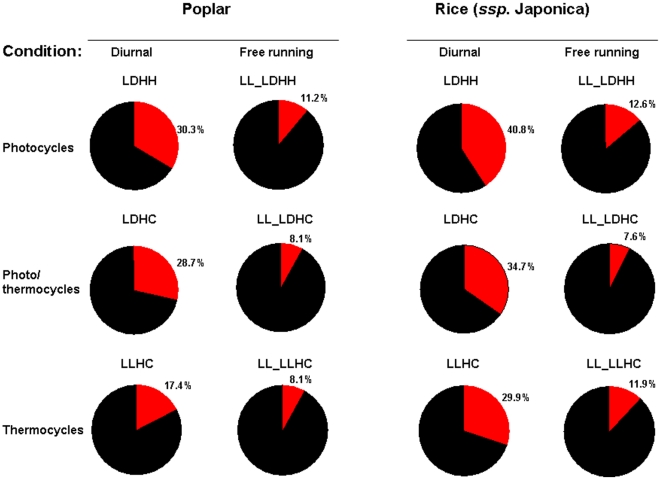
Sizable proportions of poplar and rice transcriptomes display daily oscillations in RNA abundance with peak expression encompassing all phases of the day. Proportions of poplar and rice genes rhythmically (red sector) expressed under diurnal and free running (circadian) conditions. Transcripts were considered cyclically expressed if the Pearson correlation coefficient *r* between the data and respective HAYSTACK pattern model ([1], [11]; http://haystack.cgrb.oregonstate.edu/) was 0.75 or greater. The proportions were calculated as ratios of the number of the unique cycling genes to the total number of unique gene models represented on array. Diurnal and circadian segments of each time course were separated by a spacer period of forty eight hours of continuous light as described in the Materials and Methods.

**Figure 2:**
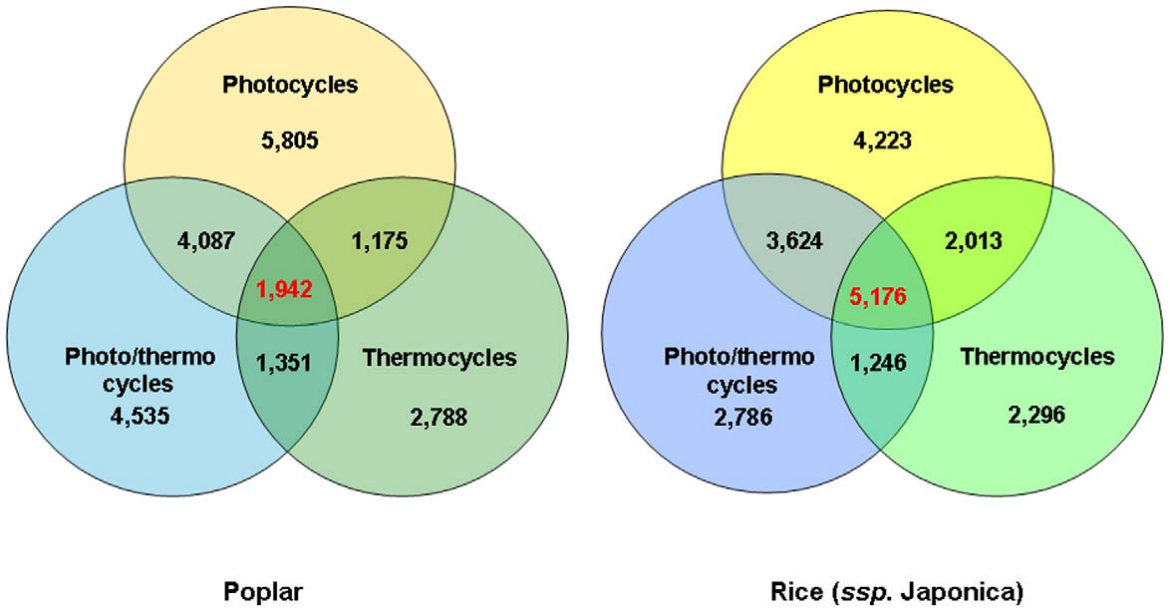
Overlap of oscillating transcripts under various driven conditions. Venn diagrams show distribution of oscillating transcripts in poplar (left) and rice (right) under photo-, thermo- and photo/thermocycles. Collectively, 20,619 and 21,364 (for poplar and rice, respectively) unique gene models were rhythmically expressed under all diurnal conditions. Of these, 1,942 poplar (11.7%) and 5,176 rice (24.6%) transcripts oscillated under all three tested diurnal conditions. To compile the lists of genes cycling under each condition, the array probe sets displaying cycling patterns of expression were matched to their respective gene models as described in the Materials and Methods.

**Figure 3:**
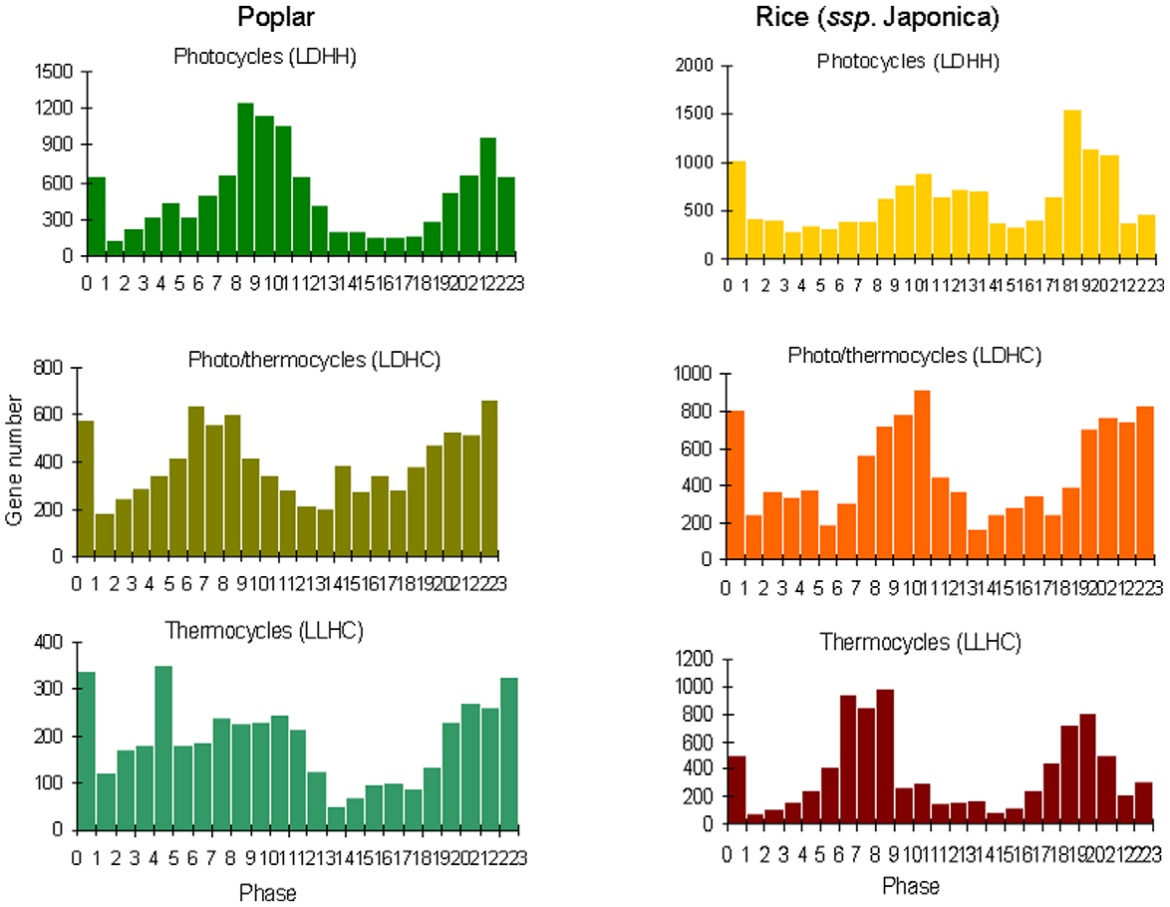
Rhythmic rice and poplar transcripts encompass all phases of the day peaking a few hours before light/dark transitions. Histograms of phase call frequency distributions among cycling poplar and rice genes according to phase of day. The distribution of the phase frequencies is broadly consistent with that of Arabidopsis plants grown under similar conditions [1]. Each phase bin corresponds to a one-hour increment.

Analyses of the resulting diurnal datasets showed that photocycles and thermocycles drove rhythmic expression of a sizable proportion of rice and poplar transcriptomes (**Figure 1**). The maximum number of rhythmic transcripts in both species was detected under driven (diurnal) conditions. All circadian (free running) conditions produced 2- to 4-fold fewer rhythmic transcripts than their respective diurnal conditions. In both rice and poplar the highest and the lowest proportions of the rhythmic transcripts under driven (diurnal) conditions were associated with photo- and thermocycles, respectively. The number of transcripts regulated by photo/thermocycles (LDHC) was intermediate as compared to photo- (LDHH) and thermocycles (LLHC) alone. Collectively, 60.9% (21,683 out of total 38,581 unique gene models) of *P. trichocarpa* and 59.5% (21,364 out of 35,928 unique gene models) of *O. sativa* ssp. *japonica* transcripts represented on arrays were expressed rhythmically under at least one diurnal condition (**Figure 2**). Using the phase calls provided by HAYSTACK algorithm we determined that rhythmic rice and poplar genes encompassed all phases of the day (**Figure 3**). Notably, a significant proportion of transcripts peaked a few hours before the light/dark transitions (dawn and dusk) resembling the bimodal distribution observed in Arabidopsis [1] and consistent with the expression of many circadian regulated genes in anticipation of the dawn and dusk light/dark transitions. The clusters of cycling genes with both high and low expression levels were evenly distributed across all phases of the day (Figure S7).

### Conserved phasing of gene expression across monocot and dicot plants

**Figure 4:**
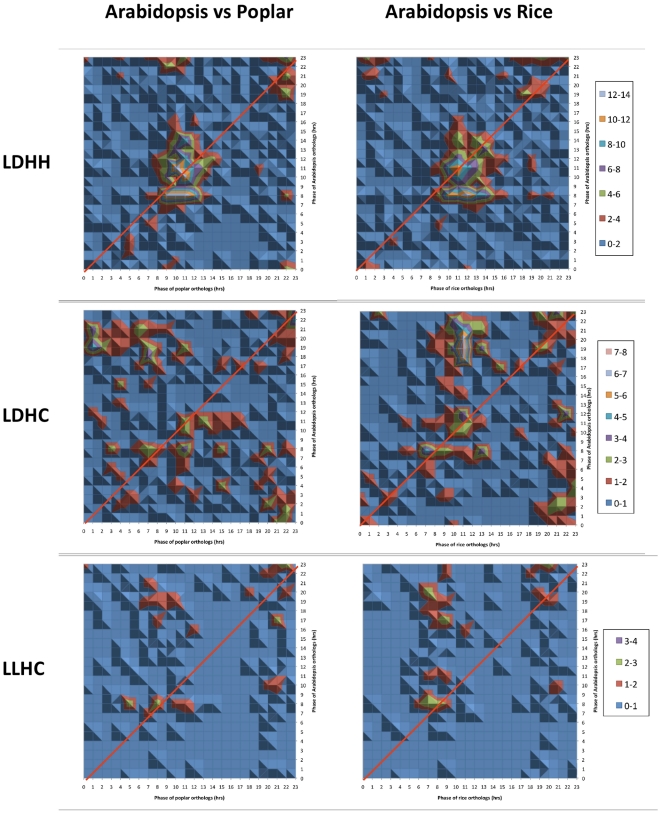
Under similar diurnal conditions Arabidopsis-rice-poplar orthologs are phased to similar times of day. 28–34% of rhythmically expressed Arabidopsis-rice-poplar orthologs were phased within three hours of each other under photo- (LDHH), thermo-(LLHC) and photo/thermocycles (LDHC). The plots depict the number of orthologs between Arabidopsis and poplar or Arabidopsis and rice for every phase of day under LDHH, LDHC and LLHC. The number of genes is represented by a color code (below). The red line represents positions of identical phases between both species being compared. Only orthologs that were present in all three species were selected in order to allow comparisons between all three species. The sum of every phase call combination between orthologs was compiled and plotted using a Microsoft Excel surface contour plot. The color scale was adjusted depending on the number of orthologs found under each condition.

To investigate whether orthologs can be phased to the same time of day under similar environmental conditions, we generated a list of 4,835 putative Arabidopsis-rice-poplar orthologs. These genes were defined as orthologous if they were found to be mutual best BLAST hits using the ORTHOMAP tool [11]. Cycling genes were identified among the list of putative orthologs and their phase calls were compared between species. Under LDHH, most of the orthologs between species had similar phases with a concentration of conserved phasing of genes peaking around dusk. In this condition (LDHH), 605 of the 4,835 orthologs were expressed and cycled in all three species and one third of these were expressed with peak phases within three hours of each other (**Figure 4**). This proportion was increased for pairwise ortholog comparisons limited to only two species (Arabidopsis-rice; Arabidopsis-poplar, rice-poplar; data not shown). Under thermocycles alone and photo/thermocycles (LLHC and LDHC, respectively), the orthologs' phases were separated into two groups: a set of orthologs expressed at the same phase of day; and another group expressed before dusk in Arabidopsis but in the morning in both rice and poplar. Collectively, a total of 41–46% (depending on the diurnal condition) of putative Arabidopsis-rice, Arabidopsis-poplar, and rice-poplar orthologs cycled under at least one diurnal condition. Among those, 21 to 36% (depending on condition) were phased within three hours of each other (data not shown).

### Rice, poplar and Arabidopsis clock-associated genes are expressed at a similar phase of day

**Figure 5:**
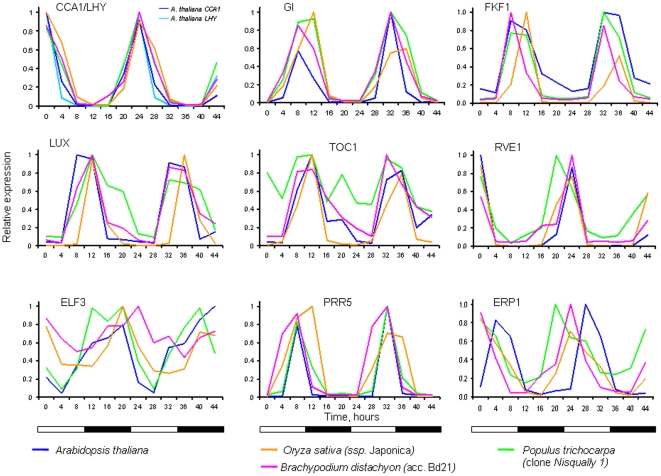
The diurnal expression profiles of the core circadian clock and several clock-associated genes are conserved among rice, poplar and Arabidopsis. Putative *O. sativa* (*ssp. japonica*), poplar, and Brachypodium clock gene orthologs were identified using criterion of the best mutual BLAST hit against the respective Arabidopsis counterparts. Time points 0 and 12 correspond to subjective dawn and dusk, respectively. Putative poplar/rice/Brachypodium orthologs correspond to Arabidopsis CCA1 (AT1G01060)/LHY (AT2G46830)/estExt_Genewise1_v1.C_LG_XIV1950/LOC_Os08g06110/Bradi3g16510, GI (AT1G22770)/estExt_fgenesh4_pg.C_LG_V1131/LOC_Os01g08700/Bradi2g05230, TOC1 (AT5G61380)/fgenesh4_pg.C_scaffold_129000038/LOC_Os02g40510/Bradi3g48880, RVE1 (At5g17300)/gw1.IV.3973/LOC_Os02g46030/Bradi3g51960, LUX (AT3G46640)/gw1.IX.4105/LOC_Os01g74020/Bradi2g62070, FKF1 (At1g68050)/estExt_fgenesh4_pg.C_LG_X0958/LOC_Os11g34460/Bradi4g16630, PRR5 (AT5G24470)/gw1.XII.1231/LOC_Os09g36220/Bradi4g36080, ELF3 (AT2G25930)/estExt_fgenesh4_pm.C_LG_VI0700/LOC_Os06g05060/Bradi2g14290), and ERP1 (At1g18330)/fgenesh4_pg.C_scaffold_122000043/LOC_Os06g51260/Bradi1g29680, respectively.

The majority of predicted poplar and rice circadian clock and clock-associated genes displayed highly conserved cycling profiles under all tested diurnal conditions (**Figure 5**). Moreover, under similar conditions (LDHH) the cycling profiles of circadian genes derived from the genome-scale microarray timecourse datasets from the monocot *Brachypodium distachyon* (Fox and Mockler, manuscript in preparation) reproducibly showed very close cycling expression patterns as well (**Figure 5**).

To further assess the conservation of expression of clock-associated genes within species, we compared the cycling profiles of clock and clock-associated genes between the *japonica* and *indica* (ArrayExpress accession number E-MTAB-275) subspecies of rice. Microarray profiles and phase calls of eight key rice clock genes [14] (*CCA1/LHY*, *GI*, *TOC1*, *RVE1*, *LUX*, *FKF1*, *PRR3*, and *PRR9*) were highly conserved in both phasing and amplitude between the *japonica* and *indica* subspecies (**Figure S2**).

### Diurnal and circadian control of genes involved in metabolic and regulatory pathways

**Figure 6:**
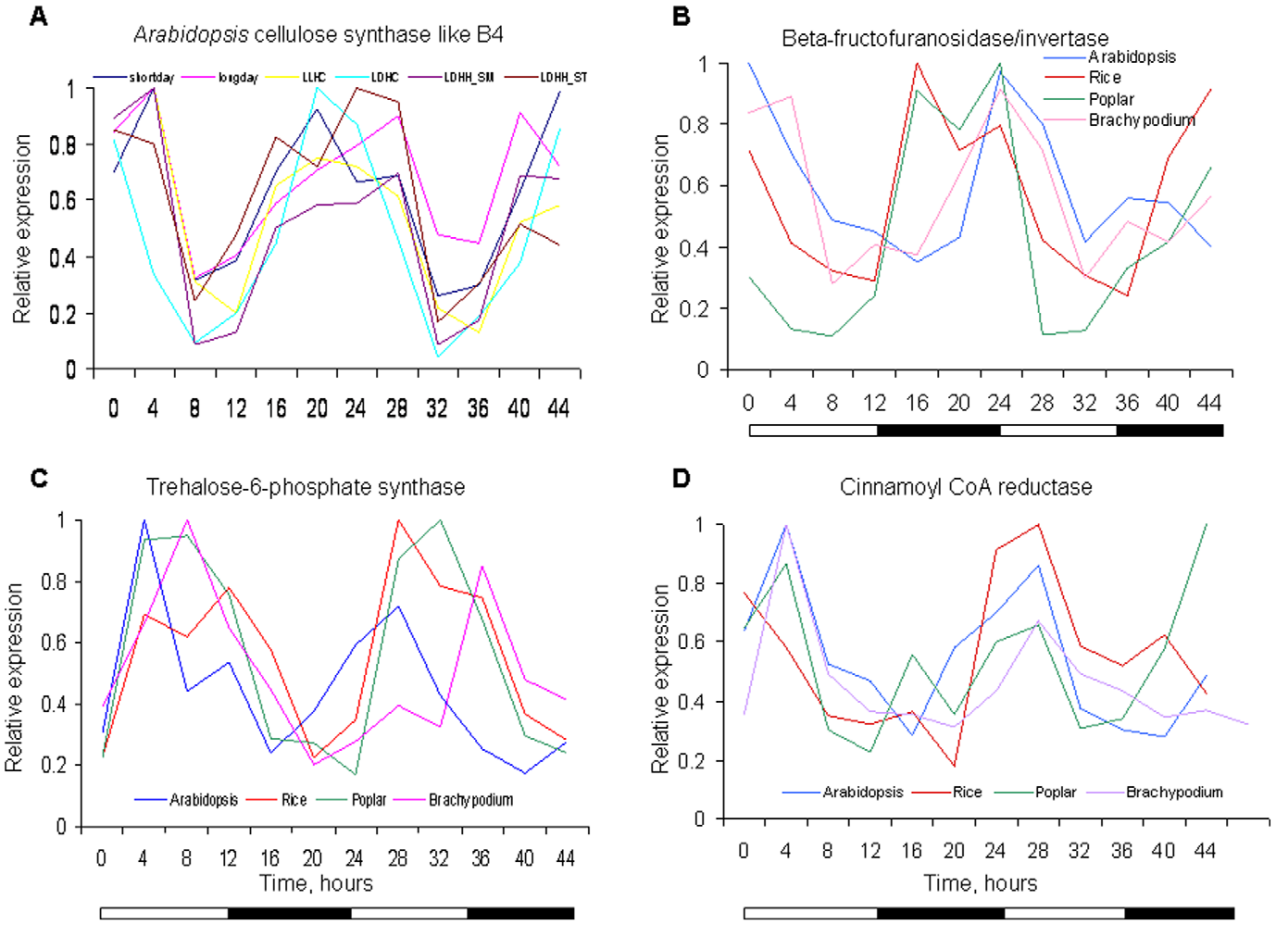
Several Arabidopsis, rice, poplar and Brachypodium orthologs are phased to similar times of day. **A**. An example of the cycling expression profile of a gene involved in cellulose biosynthesis (Arabidopsis gene AT2G32530, *CELLULOSE SYNTHASE LIKE B3*) under multiple diurnal/circadian conditions. **B**, **C**, and **D**. Expression of orthologs involved in cell wall biosynthesis in Arabidopsis, rice, poplar, and Brachypodium. **B**. Beta-fructofuranosidase/invertase, involved in sucrose catabolic process (Arabidopsis/poplar/rice, *ssp. japonica*/Brachypodium loci: - AT1G12240/estExt_fgenesh4_pg.C_LG_III0902/rice/LOC_Os04g45290/Bradi5g16900). **C**. Trehalose-6-phosphate synthase, involved in trehalose metabolism (AT1G78580/estExt_fgenesh4_pg.C_1680018/LOC_Os05g44210/Bradi2g19640). **D**. Cinnamoyl CoA reductase, involved in lignin biosynthesis (AT1G15950/estExt_fgenesh4_kg.C_LG_III0056/LOC_Os08g34280/Bradi3g36890).

**Figure 7:**
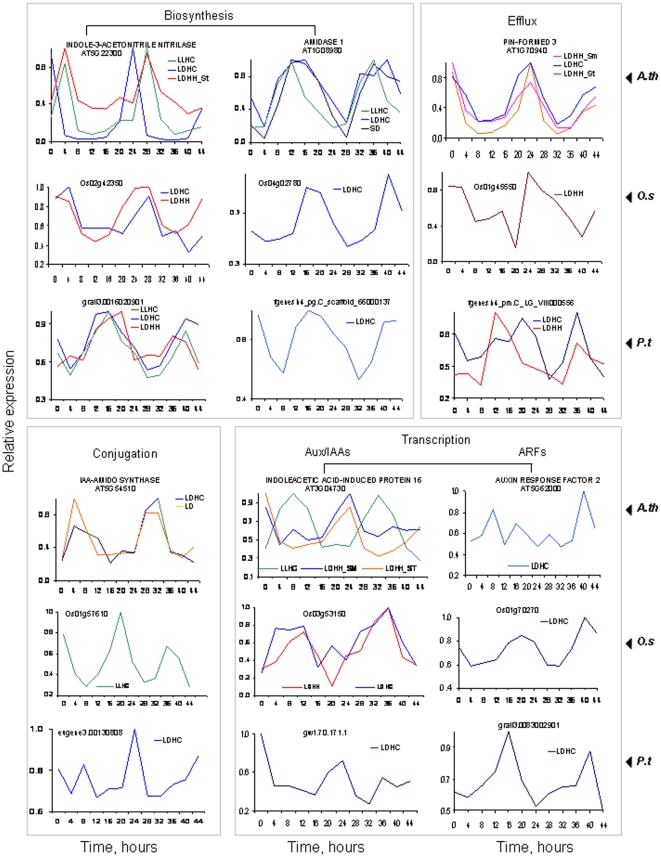
All major steps of auxin biosynthesis and response pathways in rice, poplar, and Arabidopsis include at least one cycling ortholog. Only array probe sets identified by HAYSTACK as cycling under the tested diurnal conditions are shown. Time points 0 and 12 correspond to subjective dawn and dusk, respectively. *A.th* - *Arabidopsis thaliana*, *O. sativa* - *Oryza sativa*, *ssp. japonica*, *P.t – Populus trichocarpa*, clone *Nisqally 1*.

We found many orthologs associated with specific metabolic processes with peak expression at the same time of day. For example, transcripts for several genes involved in cell wall biosynthesis cycled with strikingly conserved phasing in both dicots and monocots (**Figure 6**). Among the most broadly represented pathways were those associated with auxin, for which all steps including biosynthesis, perception, conjugation and transcriptional regulation included at least one pair of rhythmic Arabidopsis/poplar and Arabidopsis/rice orthologs (**Figure 7**). Among transcription factors implicated in auxin pathways, both the Aux/IAA and ARF families of TFs contained conserved rhythmically expressed members.

**Figure 8:**
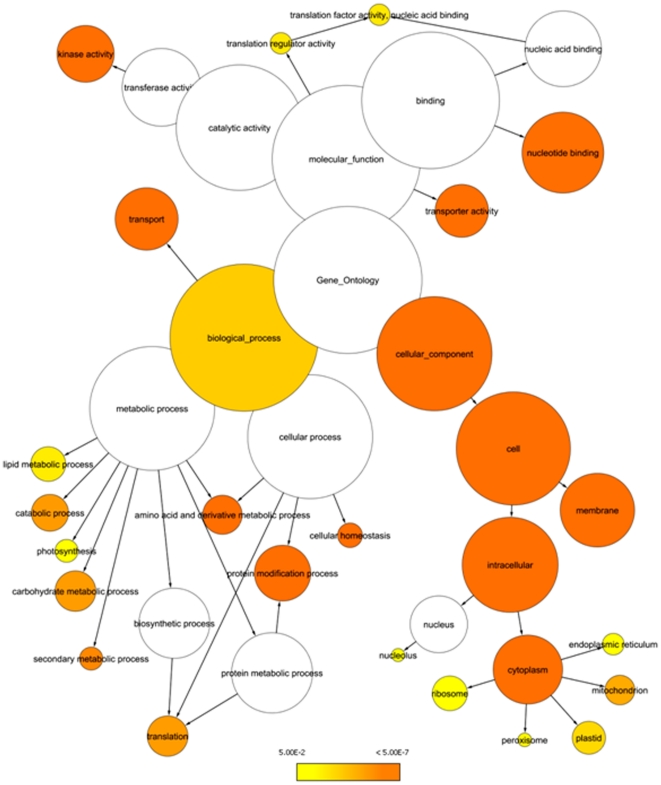
Gene ontology (GO) categories overrepresented among rice (*ssp. japonica*) cycling genes under photoperiods (LDHH). The shaded circles indicate overrepresented GO categories (categories with FDR≤0.05 are shown in yellow) based on significance level. The radius of each circle denotes the number of genes in each category. The list of cycling genes was generated using HAYSTACK with best fitting model cutoff value *r*≥0.9. GO overrepresentation graph was generated using the network visualization tool Cytoscape (http://www.cytoscape.org/).

The analysis of gene ontology (GO) categories associated with cycling genes revealed several GO terms overrepresented among oscillating rice (*ssp. japonica*) genes. Thus, under photocycles (LDHH) rice cycling genes were associated with GO categories such as lipid and carbohydrate metabolic processes, photosynthesis, nucleotide binding, translation, and amino acid and nucleotide metabolism (**Figure 8**). Under photocycles, these and several other overrepresented GO categories overlapped between rice and poplar (**Figure S3**). Mapping of oscillating genes to the 339 currently defined metabolic pathways in the RiceCyc database (http://www.gramene.org/pathway/, [15]) suggested that many of the major metabolic pathways in rice are under diurnal and/or circadian control. Under photocycles, a total of 2,078 rhythmically expressed genes mapped to defined metabolic pathways of rice (**Figure S4**). We found multiple examples of metabolic pathways that were broadly represented at every step by cycling genes, although secondary metabolic pathways showed the least representation by cycling genes (**Figure S5**).

### A large proportion of transcription factor transcripts exhibit daily rhythms in mRNA abundance

**Figure 9:**
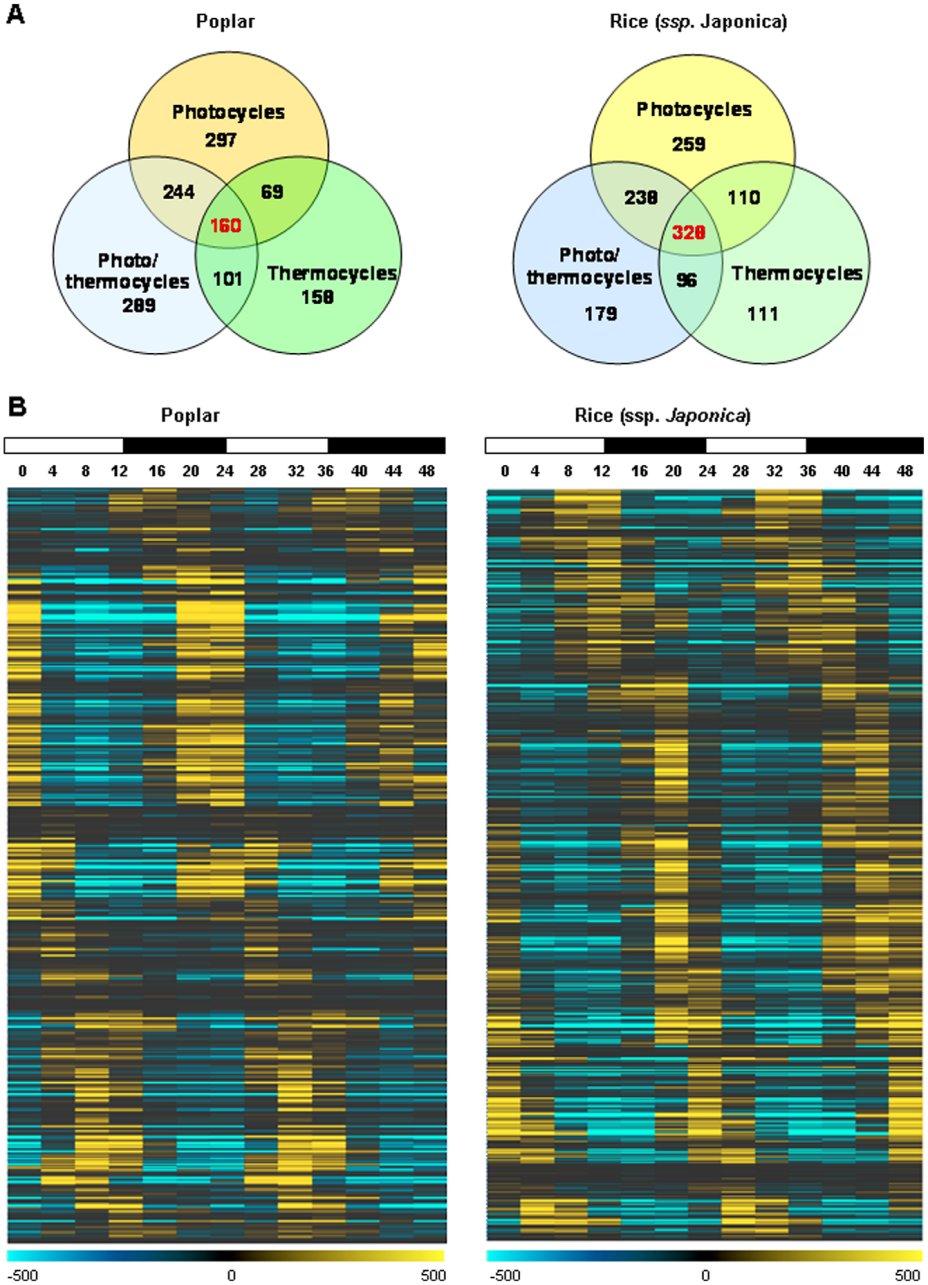
Rhythmic expression of poplar and rice transcription factor transcripts. **A**. A large portion of the *P. trichocarpa* and *O. sativa* TFs represented on arrays are rhythmically expressed under photo-, thermo-, and photo/thermocycles. **B**. Rhythmically expressed TF transcripts encompass all phases of the day peaking a few hours before light/dark transitions. Poplar and rice TFs cycling in driven condition are listed in **Table S1**. **B**. Peak expression of cycling TFs occurs at all phases of the day. Expression heat maps of *Populus trichocarpa* and *O. sativa* (*ssp. japonica*) TFs oscillating under photocyles (LDHH). Mean centered expression levels are depicted in yellow (high expression) and blue (low expression). The heat map was generated using HAYSTACK output filtered using a Pearson correlation coefficient cutoff value *r*≥0.9.

We further investigated rhythmic expression of transcription factors (TFs). For *Populus trichocarpa*, 2,052 (out of total predicted 2,576) TF genes (http://dptf.cbi.pku.edu.cn/, [16]) were represented on the Affymetrix poplar array. The *O. sativa (ssp. japonica)* microarrays represented 2,134 out of 2,384 predicted *japonica* rice TFs (http://drtf.cbi.pku.edu.cn/, [17]). Among those, 64.2% (1,318 non-redundant *P. trichocarpa* gene models) and 61.2% (1,307 non-redundant *O. sativa*, *ssp. japonica* gene models) of putative TF transcripts detectable on the microarrays cycled under at least one diurnal condition in poplar and rice, respectively. The proportions and partitioning of oscillating TFs among the different timecourse conditions was broadly consistent with the distributions among all cycling genes, i.e., the highest number of rhythmically expressed was driven by photocycles, the lowest – by thermocycles (**Figure 9A**). Several hundred genes encoding TFs displayed oscillating expression patterns under all tested diurnal conditions. Similar to the bulk of all nuclear-encoded genes, the expression profiles of cycling rice and poplar TFs showed a bimodal frequency distribution peaking at dusk and dawn (**Figure 9B**).

An examination of TF gene family representation among the 328 rice TFs that cycled robustly under all three diurnal conditions showed that the set of core cycling TFs was enriched for members of the CONSTANS-like (C2H2-CO-like; *p*<1×10^−6^), heat shock (HSF; *p*<3×10^−6^), and MYB-related (MYB; *p*<3.7×10^−5^) TF families (http://plntfdb.bio.uni-potsdam.de). To demonstrate the conservation of rhythmic expression in some TFs across species, we randomly selected a group of poplar transcripts that cycle robustly with high amplitude across all diurnal conditions. The transcript abundance profiles among these TFs represented all phases of day with many transcripts peaking at light/dark transition periods (**Figure S6**). Next, we identified putative rice orthologs for this group of genes based on mutual best BLAST hits. Interestingly, predicted rice orthologs also showed robust cycling under photocycles (**Figure S6**) peaking at a similar phase (within three hours) as their respective poplar counterparts, consistent with deep conservation of transcriptional regulatory networks among mono- and dicotyledonous species.

### Conserved *cis*-regulatory elements in promoters of co-expressed genes

**Figure 10:**
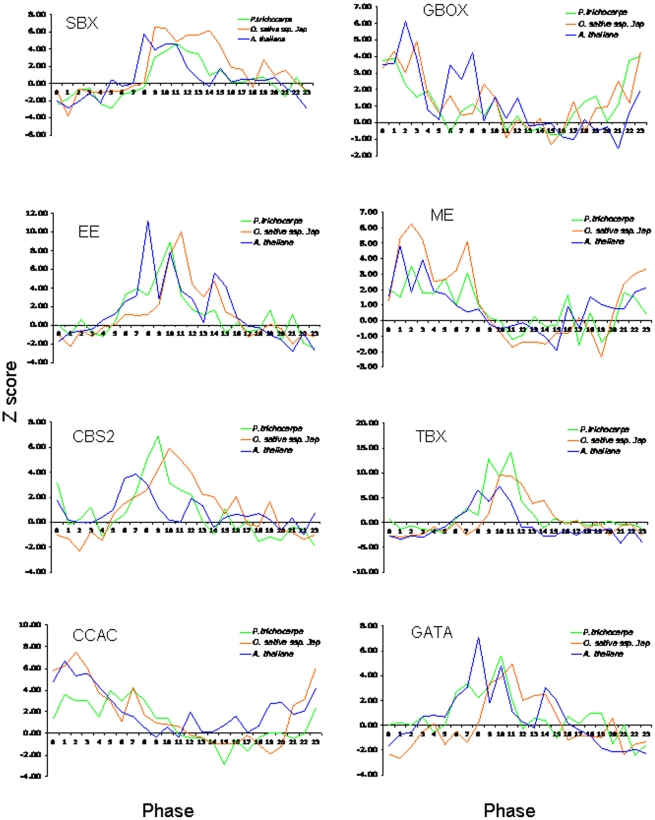
Identification of conserved *cis*-elements in promoters of Arabidopsis, rice and poplar genes cycling in the LDHH condition. ELEMENT-based enumerative promoter analysis and Z-score profile comparisons between rice, poplar and Arabidopsis. Z-score profiles were summarized for diurnal and circadian associated elements exhibiting conserved time-of-day overrepresentation across rice, poplar and Arabidopsis. A Z-score cutoff threshold value 2.33 corresponding to a *p*-value of 0.01 was selected arbitrarily. Time points 0 and 12 correspond to subjective dawn and dusk, respectively.

To identify *cis*-regulatory elements associated with diurnal/circadian regulation of rice and poplar gene expression, as well as motifs conserved with those in Arabidopsis [1], we mined the promoters of cycling poplar, rice, and Arabidopsis genes to identify DNA elements that could be associated with expression at a specific time of day. Using the HAYSTACK algorithm we identified clusters of co-expressed cycling genes in rice, poplar, and Arabidopsis. The putative promoters (500 base pairs upstream of the annotated gene model) for genes in each phase bin were searched for overrepresented 3–8-mer promoter elements (or words) using ELEMENT [1]. The resulting Z-scores were assembled into Z-score profiles corresponding to the 24 1-hour phases of the day and these Z-score profiles were further analyzed to identify profiles in which multiple consecutive Z-scores exceeded a threshold corresponding to a 5% false-discovery rate. The Z-score profiles were also compared between species using HAYSTACK to identify promoter elements with conserved enrichment profiles. This analysis revealed conserved diurnal- and circadian-associated promoter elements that were overrepresented among co-expressed genes in rice, poplar, and Arabidopsis at specific times of day (**Figure 10**). Motifs representing all phases of the day included the Morning Element (ME) [2], [18], Evening Element (EE) [2], GBOX [2], [19], [20], [21], GATA motif [22], [23], CBS [24], and SBX/TBX [1].

## Discussion

Photocycles and/or thermocycles drive oscillations of a large proportion of expressed nuclear genes in rice and poplar. The maximum number of rhythmically expressed transcripts in both species was detected under driven (diurnal) conditions while free running conditions were associated with fewer, circadian-regulated, cycling transcripts. The peak phases of expression for cycling transcripts represented all phases of the day, with the greatest numbers of cycling genes exhibiting peak expression phased to a few hours before dusk and dawn, consistent with anticipation of the light/dark transitions.

Approximately 60% of the rice and poplar transcripts represented on the microarrays used in this study displayed rhythmic expression in at least one of the six tested diurnal/circadian time courses. This observation is broadly consistent with earlier studies [2], [4], [5], [25], [26] estimating that at least 10–30% of the expressed genes in Arabidopsis are circadian regulated as well as more recent studies [1], [11] indicating that under a broad set of environmental conditions (a total of eleven diurnal/circadian time courses) up to 89% of transcripts in Arabidopsis can display rhythmic expression patterns. Because our current survey was limited to fewer diurnal/circadian conditions, it is likely that the number of cycling transcripts in rice and poplar could be significantly higher under more diverse environmental conditions.

The major distinctions regarding rhythmic gene expression patterns in rice and poplar as compared to Arabidopsis [1], [11] included a lower proportion of transcripts that cycle under circadian (free running) conditions. This apparent discrepancy can, in part, be explained by two major differences in the experimental design. First, to decrease residual diurnally driven oscillations and to identify the most robust cyclers, we provided a spacer period comprising 48 hours of continuous conditions between the diurnal sampling and the circadian sampling segments of the time courses. In contrast, such a spacer segment was not included in the Arabidopsis time courses [1]. Since in the latter studies, the first light period is preceded by a dark period, it is thus indistinguishable from a regular light/dark transition and could be driving expression of some genes in a non-circadian fashion. We reason that a spacer period preceding the free running time course could thus contribute to the apparent decreased proportion of circadian cycling transcripts in rice and poplar because of the expected dampening of the circadian harmonics and reducing amplitude of the oscillations, which occurs under continuous light or dark conditions [9]. Second, for both poplar and rice we used high fluence light (but within the physiologically acceptable range). The intensity of ambient light can influence the pace at which the clock runs in constant conditions and in many light-active organisms including plants, higher fluences of continuous light shorten the free running period [27]. This phenomenon could have changed the period of some genes to such an extent that they escaped our analysis.

For each condition tested, there were cycling genes with peak expression at each phase of day. The abundance of cycling genes whose expression was phased to just prior to dawn and dusk compared to other times of the day was consistent with similar bimodal profiles of cycling gene abundance observed in Arabidopsis [1]. This observation supports a general notion that the plant circadian clock correctly matches physiological processes to the external environmental transitions. In turn, anticipation of evening and morning environmental changes provides a competitive advantage and increases organism fitness [26], [28].

In higher plants, the circadian clock is regulated by at least three interconnected transcriptional regulatory feedback loops (reviewed in [7]). The central oscillator loop is formed by the CCA1/LHY, TIMING OF CAB EXPRESSION 1 (TOC1) and CCA1 HIKING EXPEDITION (CHE) transcription factors [8], [10], [29]. CCA1 and TOC1 reciprocally regulate each other's expression and peak in the morning and evening phases, respectively [8], [10], [29]. The clock integrates environmental inputs such as light and temperature while output circuitry regulates physiological processes through an expansive regulatory network of transcription factors. Consistent with this model, a large proportion of cycling transcripts in Arabidopsis, rice and poplar peaked at dusk and dawn. Interestingly, a similar trend was observed among the transcription factors of all three species. The finding that large proportions of transcription factors in plants exhibit rhythmic expression patterns is consistent with extensive diurnal and circadian regulation and plasticity of transcriptional circuits and biological networks.

The expression patterns of most clock and clock-associated genes were remarkably similar among both mono- (rice and Brachypodium) and dicotyledonous (Arabidopsis and poplar) species suggesting strong conservation of circadian networks in higher plants. This similarity was even more striking among the rice subspecies. The oscillation profiles and phase calls among *O. sativa* subspecies *japonica* and *indica* were nearly indistinguishable under the tested conditions.

Under photocycles alone, 28–34% of the three-species (Arabidopsis-rice-poplar) orthologs were co-expressed with phases within three hours of each other, suggesting strong conservation of phased expression for some transcriptional circuits. In contrast, under diurnal conditions with a temperature component (thermocycles and photo/thermocycles) another subset of orthologs was expressed before dusk in Arabidopsis but in the morning in both rice and poplar. This phenomenon may reflect a difference in temperature sensitivity or perception between Arabidopsis, poplar and rice. Temperature is an important environmental time cue and plants can be entrained to temperature cycles. Moreover, the transcription of genes in Arabidopsis can be regulated by two different circadian clocks distinguishable by their sensitivity to environmental temperature signals [30]. Although we found many examples of phase-conservation among rice, Arabidopsis, and poplar transcription factor orthologs, in other instances Arabidopsis-rice-poplar orthologs were phased to different times of day (data not shown) suggesting species-specific diversification of some diurnal/circadian-associated transcriptional circuits.

GO analysis confirmed that from a signaling perspective, light, temperature and the circadian clock have deep regulatory ramifications for almost all pathways in plants including transcriptional regulation, hormone signaling, and metabolic pathways. Strong conservation of circadian phasing among most clock genes and many transcription factors suggests that circadian-regulated transcriptional networks play a major role in plant adaptation to daily and seasonal environmental changes.

We found many instances of rice-poplar-Arabidopsis orthologs involved in the same metabolic process or reaction that were phased to the same time of day in all three species. These examples demonstrate a high degree of conservation of circadian regulation for some metabolic pathways among di- and monocotyledonous species. We found examples of diurnal and circadian regulated rice-poplar-Arabidopsis orthologs and homologs at each step of auxin pathways including biosynthesis, efflux, perception and transcriptional regulation (for both Aux/IAA and ARF TFs). Our findings in rice and poplar are broadly consistent with the results from others that circadian regulated genes involved in auxin signaling are disproportionately overrepresented in Arabidopsis [4], [31].

Closer examination of functional annotations revealed that several GO categories were overrepresented among oscillating genes. The major overrepresented GO categories included photosynthesis, lipid and carbohydrate metabolic processes, nucleotide binding, translation, and amino acid and nucleotide metabolism. Moreover, these and other categories broadly overlapped between rice and poplar. Conversely, mapping cycling genes to rice metabolic pathways suggested that many metabolic pathways are saturated with circadian regulated genes. Altogether, these findings confirm conservation of the circadian control of at least several main metabolic pathways among mono- and dicotyledonous plants.

Even though large-scale nucleotide sequence homology over the lengths of promoters of orthologous Arabidopsis, rice and poplar genes can be very limited (data not shown), we were able to identify conserved short 3–8-mer *cis*-elements overrepresented in promoters of specific sets of co-expressed genes. Out study confirmed the importance of previously known and uncharacterized promoter motifs in diurnally regulated gene expression that were predicted to be conserved in prior *in silico* analyses [1], [32]. One of the *cis*-elements, the PBX motif (ATGGGCC), representing the midnight regulatory module (PBX/TBX/SBX) has been shown to be sufficient to drive diurnal and circadian condition-dependent expression of a luciferase reporter in transgenic Arabidopsis seedlings [1]. Notably, the GBOX Z-score profile was more “noisy” than the profiles of other *cis*-elements. Considering the high frequency of occurrence of the GBOX in plant genomes and its ubiquitous regulatory nature (reviewed in [33], [34]) this observation was unsurprising. However, the statistical prevalence of the GBOX at morning hours over background was nevertheless evident. Identification of orthologous *cis*-regulatory motifs in rice and poplar promoters further strengthens the notion that the diurnal/circadian regulatory network is strongly conserved among mono- and dicotyledonous plants. Moreover, the confirmation of the earlier *in silico* predictions of diurnal/circadian regulatory circuitry in rice and poplar validates the bioinformatic methods employed and suggests that the approach could be utilized to find other transcriptional regulatory modules in plants.

In summary, using a combination of genomics and bioinformatics tools, we have demonstrated the profound conservation of the transcriptional regulatory network in dicot and monocot plants that allows global adaptation to the ever-changing daily environment. Further studies of the conservation and diversification of diurnal and circadian regulated pathways among different plant phyla will ultimately facilitate engineering of plants with increased fitness for diverse environments.

## Materials and Methods

### Plant material, growth conditions and time course sampling

Rice (*Oryza sativa*, *ssp. japonica*, cultivar Nipponbare 1 and *ssp. indica*, cultivar 93-11) and poplar (*Populus trichocarpa*, clone *Nisqually*-1) plants were grown in a Conviron PGR15 growth chamber using precise control of temperature, light, and humidity.

Three-month-old rice plants were entrained for at least one week under the respective conditions prior to initiation of each experiment. Leaves and stems from individual rice plants were collected every four hours for 48 hrs followed by a two day free running spacer period. The sampling continued for another 48 hours under the same free running conditions. Diurnal rice conditions included three combinations of 12 hours light (L)∶12 hours dark (D) cycles and 31°C/20°C thermocycles (**Figure S1**). These were: photocycles (LDHH), 12L∶12D at constant temperature (31°C; HH); photo/thermocycles (LDHC): 12L∶12D with a high day temperature (31°C) and a low night temperature (20°C); and thermocycles (LLHC): continuous light (LL) with 12 hours high: 12 hours low temperature (31°C, day; 20°C, night). Light intensity and relative humidity were 1000 µmol m^−2^ s^−1^ and 60%, respectively. Circadian (free-run) conditions were under continuous light and constant high temperature (31°C).

Three-month-old poplar (*Populus trichocarpa*, clone *Nisqually-1*) plants grown from fresh cuttings were entrained for at least one week under the respective conditions prior to initiation of each experiment. Young poplar leaves (including petioles) were collected every four hours for 48 hrs in diurnal (driven) conditions followed by a two day free running spacer period. The sampling continued for another 48 hours under the same free running conditions. Diurnal (driven) conditions for poplar were: 12L∶12D light cycles and 25°C∶12°C thermocycles in three different combinations. These were: photocycles (LDHH), 12L∶12D at a constant temperature (25°C; HH); photo/thermocycles (LDHC): 12 hours light (L)∶12 hours dark (D) with a high day temperature (25°C) and a low night temperature (12°C); and thermocycles (LLHC): continuous light (LL) with 12 hours high∶12 hours low temperature (25°C, day; 12°C, night). Light intensity and relative humidity were 700 µmol m^−2^ s^−1^ and 50%, respectively. Circadian (free-run) conditions were under continuous light and constant high temperature (25°C).

### RNA preparation, cRNA synthesis, and microarray hybridization

Leaf tissues were pulverized in liquid nitrogen. Total cellular RNA was extracted and treated with RNase-free DNase essentially as previously described [35]. 2 µg of total RNA was used to generate biotinylated complementary RNA (cRNA) for each treatment group using the One-Cycle Target Labeling protocol (Affymetrix, Santa Clara, CA) from the GeneChip® Expression Analysis Technical Manual (701021 Rev. 5). Isolated total RNA was checked for integrity and concentration using the RNA 6000 Nano LabChip kit on the Agilent Bioanalyzer 2100 (Agilent Technologies, Inc., Palo Alto, CA). RNA was reverse transcribed using a T7-(dT) 24 primer and Superscript II reverse transcriptase (Invitrogen, Carlsbad, CA) and double stranded cDNA was synthesized and purified with GeneChip® Sample Cleanup Modules (Affymetrix, Santa Clara, CA). Biotinylated cRNA was synthesized from the double stranded cDNA using T7 RNA polymerase and a biotin-conjugated pseudouridine containing nucleotide mixture provided in the IVT Labeling Kit (Affymetrix, Santa Clara, CA). Prior to hybridization, the cRNA was purified with GeneChip® Sample Cleanup Modules (Affymetrix, Santa Clara, CA), and fragmented. 10 µg from each experimental sample along with Affymetrix eukaryotic hybridization controls were hybridized for 16 hours to Affymetrix poplar or rice genome arrays in an Affymetrix GeneChip® Hybridization Oven 640. An Affymetrix GeneChip® Fluidics Station 450 was used to wash and stain the arrays with streptavidin-phycoerythrin (Molecular Probes, Eugene, OR), biotinylated anti-streptavidin (Vector Laboratories, Burlingame, CA) according to the standard antibody amplification protocol for eukaryotic targets. Arrays were scanned with an Affymetrix GeneChip® Scanner 3000 at 570 nm. The Affymetrix eukaryotic hybridization control kit and Poly-A RNA control kit were used to ensure efficiency of hybridization and cRNA amplification. All cRNAs from each 13-time point batch were synthesized at the same time. Hybridizations were conducted with one replicate for each time point. Each array image was visually screened to identify signal artifacts, scratches or debris. Detailed protocols described above are available at http://corelabs.cgrb.oregonstate.edu/protocols.

### Data Analysis

#### Array quality control and normalization

Standard Affymetrix quality control procedures were performed using the BioConductor packages simpleaffy and affyPLM (www.r-project.org). Data manipulations were performed using R, a language and environment for statistical computing (www.r-project.org, [36], Perl, and MySQL. Arrays were normalized and expression estimates for each time point were calculated using the RMA algorithm [37].

#### Identification of cycling genes and co-expression analysis

Expression level time series were assembled and cycling genes were identified using the HAYSTACK algorithm [1], [11]. HAYSTACK calculates a Pearson correlation value between the model series values and the expression data series values for each probe set. This calculation is amplitude-independent, allowing for comparisons between the model series and any gene expression profile, regardless of the expression level. All microarray datasets were assembled into a public database of diurnal and circadian regulated genes in rice (*Oryza sativa*, both *ssp. japonica* and *indica*), *Populus*, and Arabidopsis (http://www.diurnal.cgrb.oregonstate.edu). Gene Spring 7.3 (Agilent Technologies, USA), software was used for further data analysis of HAYSTACK output and graphical display of results. Pattern analysis of gene expression data was performed using a hierarchical centroid-clustering algorithm with average linkage and within-gene averaging and scaling.

#### Functional analysis, protein comparisons, ortholog predictions

Functional enrichment/overrepresentation analysis was carried out using the network visualization program Cytoscape with GO plugins. For over representation determination, the Benjamini and Hochberg FDR-adjusted significance level cutoff was set at 0.05. The circles (**Figure 9** and **Figure S5**) were shaded based on significance level (yellow indicates FDR<0.05), and the radius of each circle denotes the number of genes in each category. Arabidopsis-rice, Arabidopsis-poplar, and poplar-rice orthologs were predicted using a mutual best BLAST match approach using ORTHOMAP (http://orthomap.cgrb.oregonstate.edu/) as described [38]. Three-way Arabidopsis-rice-poplar mutual orthologs were predicted using three-way mutual best BLAST matches. Phase calls for three-way orthologs were determined by querying the Diurnal database (http://diurnal.cgrb.oregonstate.edu/). The lists of predicted Arabidopsis-poplar-rice orthologs generated for each condition were further selected based on phase calls falling within a three hours range for all three orthologous genes. The lists of putative rice (*Oryza sativa*, *ssp. japonica*) TFs was obtained from The Database of Rice Transcription Factors (DRTF; http://dptf.cbi.pku.edu.cn/, [17]). The list of predicted poplar (*Populus trichocarpa*) TFs was obtained from Database of Poplar Transcription Factors (http://dptf.cbi.pku.edu.cn/, [16]). Brachypodium orthologs were determined using best mutual blast hits and Brachypodium genomic sequence [39].

#### Detection of conserved motifs in promoter sequences

To determine overrepresentation of a putative *cis*-regulatory elements (i.e. motifs, words), the ELEMENT algorithm (http://element.cgrb.oregonstate.edu [1], [11]) calculates and scores the frequency of occurrences of the specific word among promoters of genes co-expressed at the same phase of day. The Z-score profiles associated with specific motifs were generated using the following method. A Z-score was calculated for each motif, for each phase of day, using the ELEMENT tool and the sets of genes co-expressed in each phase-bin (corresponds to a one hour increment). For each 3–8-mer motif, this generated 24 discrete Z-scores. To generate a continuous enrichment profile the Z-scores for each motif were plotted over the phases of the day. To select profiles with phase-dependent enrichment, the profiles were computationally filtered as follows. Only Z-score profiles which had three consecutive phases of significant enrichment was selected. A ‘significant enrichment’ in a single phase was determined if the Z-score value was equal or greater than 2.33.

#### Datasets and accession numbers

Genome scale data for rice, poplar and Arabidopsis can be analyzed and downloaded using DIURNAL portal at http://diurnal.cgrb.oregonstate.edu. Rice and poplar array datasets are deposited in the ArrayExpress database (http://www.ebi.ac.uk/miamexpress/) under the accession numbers E-MEXP-2506 (*ssp. japonica*) and E-MTAB-275 (*ssp. indica*) for rice and E-MEXP-2509 for poplar.

## Supporting Information

Figure S1 Plant growth conditions and time course sampling strategy. Rice plants (both *ssp. japonica*, cultivar Nipponbare1 and *ssp. indica*, cultivar 93-11) were grown under the following diurnal conditions: LDHH: 12 hours light (L)/12 hours dark (D) at a constant daytime temperature (31°C; HH); LDHC: 12 hours light (L)/12 hours dark (D) at high (day)/low (night) physiological range temperature (31°C, day, 20°C, night); LLHC: continuous light (LL) for 24 hours and high/low temperature (31°C, day, 20°C, night). LL_LXLX represents LL_LDHH, LL_LDHC, or LL_LLHC. Light intensity was 1000 umol m^−2^ s^−1^; and relative humidity 60%. Poplar growth conditions were similar to those described for rice, except the light intensity was 700 umol m^−2^ s^−1^ and the temperature was 25°C (day) and 12°C (night).
(TIF)

Figure S2 Diurnal expression profiles of circadian clock genes are highly conserved between *japonica* and *indica* subspecies of O. sativa. Microarray profiles of the predicted rice orthologs of Arabidopsis circadian genes *CCA1 (CIRCADIAN CLOCK ASSOCIATED 1), GI (GIGANTEA), TOC1 (TIMING OF CAB EXPRESSION 1), RVE1 (REVEILLE 1), LUX (LUX ARRHYTHMO), FKF1 (FLAVIN-BINDING, KELCH REPEAT, F BOX 1), PRR3 (PSEUDO-RESPONSE REGULATOR 3), and PRR9 (PSEUDO-RESPONSE REGULATOR 9)*.
(TIF)

Figure S3 Gene ontology categories overrepresented among *Populus trichocarpa* cycling genes (LDHH photocycles). The yellow shaded circles represent overrepresented GO categories (FDR≤0.05). The radius of each circle denotes the number of genes in each category. The list of cycling genes was generated using HAYSTACK with a Pearson correlation coefficient cutoff value *r*≥0.9.
(TIF)

Figure S4 An overview of circadian-regulated genes mapped to major metabolic pathways in rice (*ssp. japonica*). **A**. 2,078 rhythmic genes mapped to metabolic pathways under photocycles (LDHH condition, *r*≥0.85). Black dots indicate compounds within pathways. Reactions with cycling genes are colored in red. Pathways with predominant mappings of cycling genes such as amino acid metabolism, nucleotide metabolism, and lipid metabolism are circled.
(TIF)

Figure S5 Expanded view of several pathways saturated with diurnally regulated genes. Cycling genes are depicted in red.
(TIF)

Figure S6 An example of the rhythmic expression among predicted poplar-rice TF orthologs. The 41 poplar TFs were arbitrarily selected based on high amplitude of oscillation and robust cycling under all tested diurnal conditions. Eleven out of 41 poplar TFs were identified as putative rice orthologs. The poplar TFs and corresponding rice orthologs show robust cycling profiles that encompass most of the phases of the day in the LDHH condition. Note clusters of TFs peaking at the light (L)/dark (D) transitions (dawn and dusk). Gene models corresponding to the predicted *Populus trichocarpa* and *Oryza sativa* (*ssp. japonica*) TFs were identified using the Databases of Poplar and Rice Transcription Factors (http://dptf.cbi.pku.edu.cn/).
(TIF)

Figure S7 Peak expression of circadian-regulated genes occurs at all phases of the day. Expression heat maps of poplar (*Populus trichocarpa*) and rice (*Oryza sativa*, *ssp. japonica*) transcripts oscillating under photocyles (LDHH). Note even distribution of the clusters of peaking genes across all times of day. Mean centered expression levels are shown in yellow (high expression) and blue (low expression). The input gene list was generated using HAYSTACK with a Pearson correlation coefficient cutoff value *r*≥0.9.
(TIF)

Table S1 The lists of common and unique *Oryza sativa* (*ssp. japonica*) TFs cycling under all tested diurnal conditions.
(XLS)

Table S2 Summary of *Oryza sativa* (*ssp. japonica*) GO category enrichment under photocyles (LDHH).
(PDF)
