# Supplementary figures and images for: Global Profiling of Rice and Poplar Transcriptomes Highlights Key Conserved Circadian-Controlled Pathways and cis-Regulatory Modules
Source: PLoS One. 2011 Jun 9;6(6):e16907. doi: 10.1371/journal.pone.0016907 (PMC3111414; doi:10.1371/journal.pone.0016907)

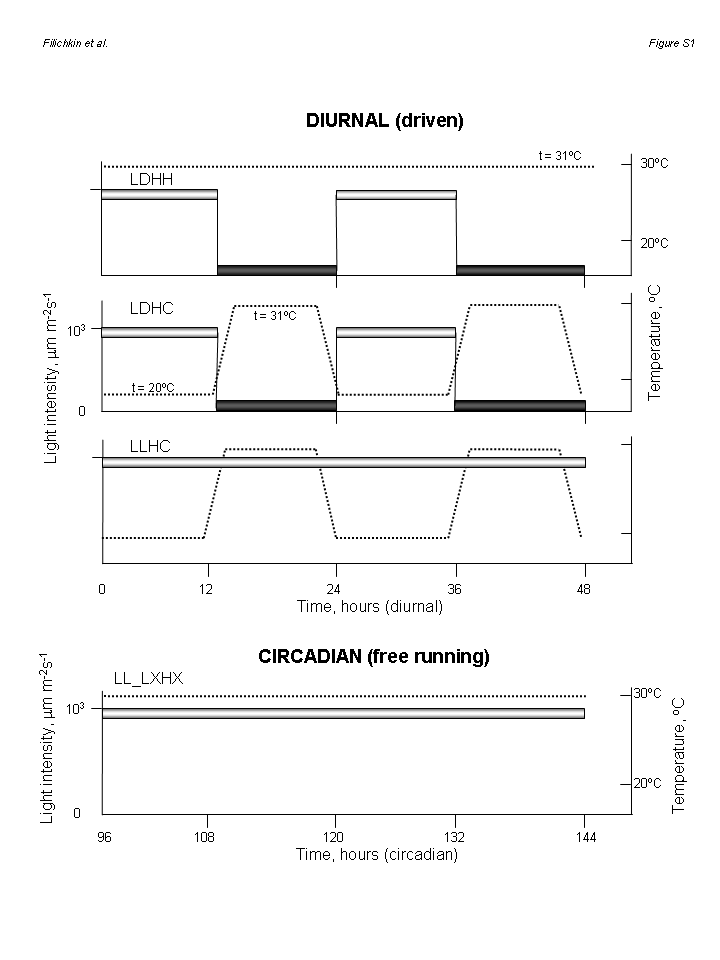

Supplement: Figure S1 — Plant growth conditions and time course sampling strategy. Rice plants (both ssp. japonica, cultivar Nipponbare1 and ssp. indica, cultivar 93-11) were grown under the following diurnal conditions: LDHH: 12 hours light (L)/12 hours dark (D) at a constant daytime temperature (31°C; HH); LDHC: 12 hours light (L)/12 hours dark (D) at high (day)/low (night) physiological range temperature (31°C, day, 20°C, night); LLHC: continuous light (LL) for 24 hours and high/low temperature (31°C, day, 20°C, night). LL_LXLX represents LL_LDHH, LL_LDHC, or LL_LLHC. Light intensity was 1000 umol m−2 s−1; and relative humidity 60%. Poplar growth conditions were similar to those described for rice, except the light intensity was 700 umol m−2 s−1 and the temperature was 25°C (day) and 12°C (night). (TIF) [file pone.0016907.s001.tif]

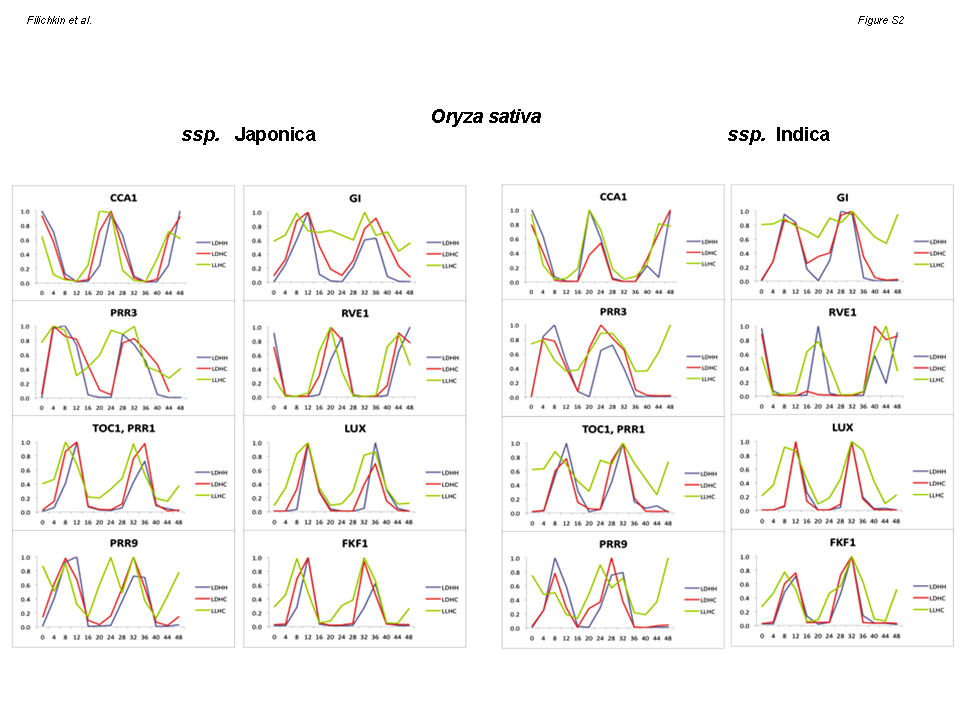

Supplement: Figure S2 — Diurnal expression profiles of circadian clock genes are highly conserved between japonica and indica subspecies of O. sativa. Microarray profiles of the predicted rice orthologs of Arabidopsis circadian genes CCA1 (CIRCADIAN CLOCK ASSOCIATED 1), GI (GIGANTEA), TOC1 (TIMING OF CAB EXPRESSION 1), RVE1 (REVEILLE 1), LUX (LUX ARRHYTHMO), FKF1 (FLAVIN-BINDING, KELCH REPEAT, F BOX 1), PRR3 (PSEUDO-RESPONSE REGULATOR 3), and PRR9 (PSEUDO-RESPONSE REGULATOR 9). (TIF) [file pone.0016907.s002.tif]

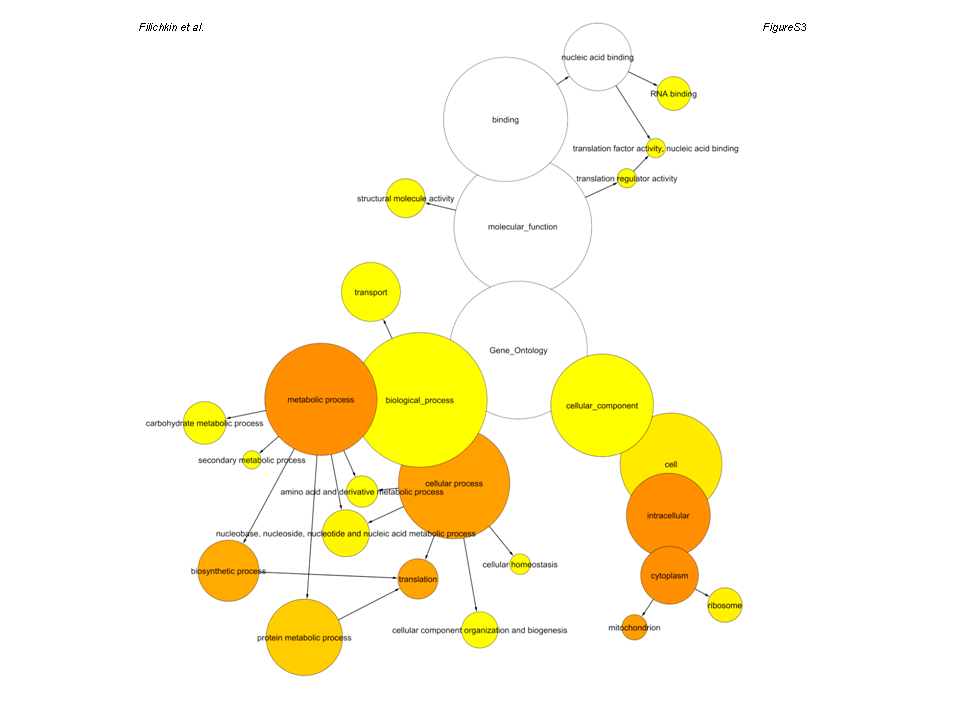

Supplement: Figure S3 — Gene ontology categories overrepresented among Populus trichocarpa cycling genes (LDHH photocycles). The yellow shaded circles represent overrepresented GO categories (FDR≤0.05). The radius of each circle denotes the number of genes in each category. The list of cycling genes was generated using HAYSTACK with a Pearson correlation coefficient cutoff value r≥0.9. (TIF) [file pone.0016907.s003.tif]

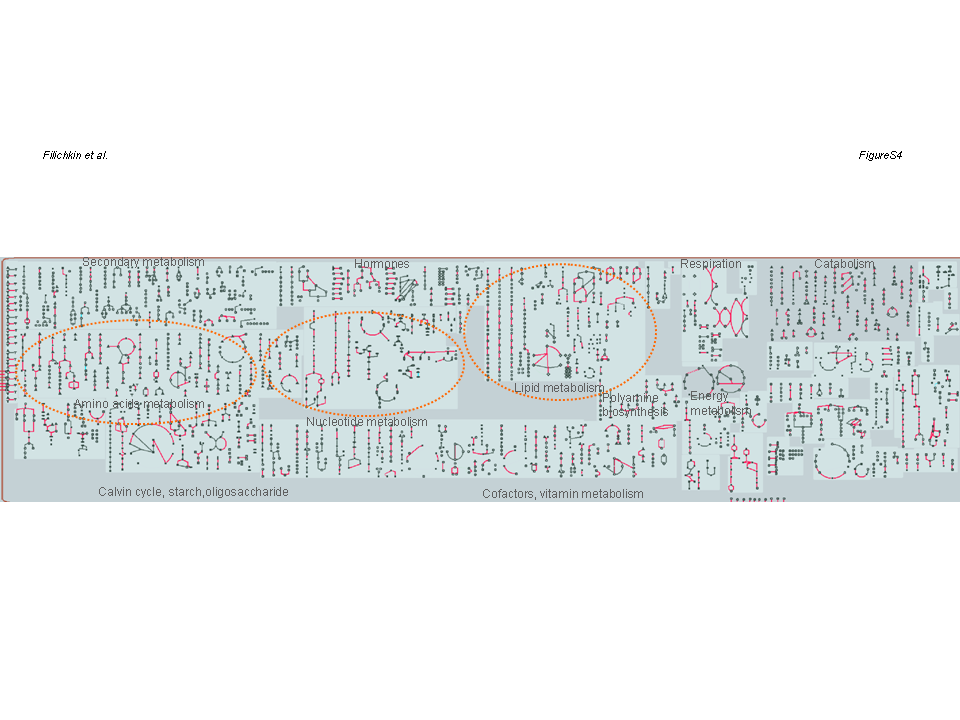

Supplement: Figure S4 — An overview of circadian-regulated genes mapped to major metabolic pathways in rice (ssp. japonica). A. 2,078 rhythmic genes mapped to metabolic pathways under photocycles (LDHH condition, r≥0.85). Black dots indicate compounds within pathways. Reactions with cycling genes are colored in red. Pathways with predominant mappings of cycling genes such as amino acid metabolism, nucleotide metabolism, and lipid metabolism are circled. (TIF) [file pone.0016907.s004.tif]

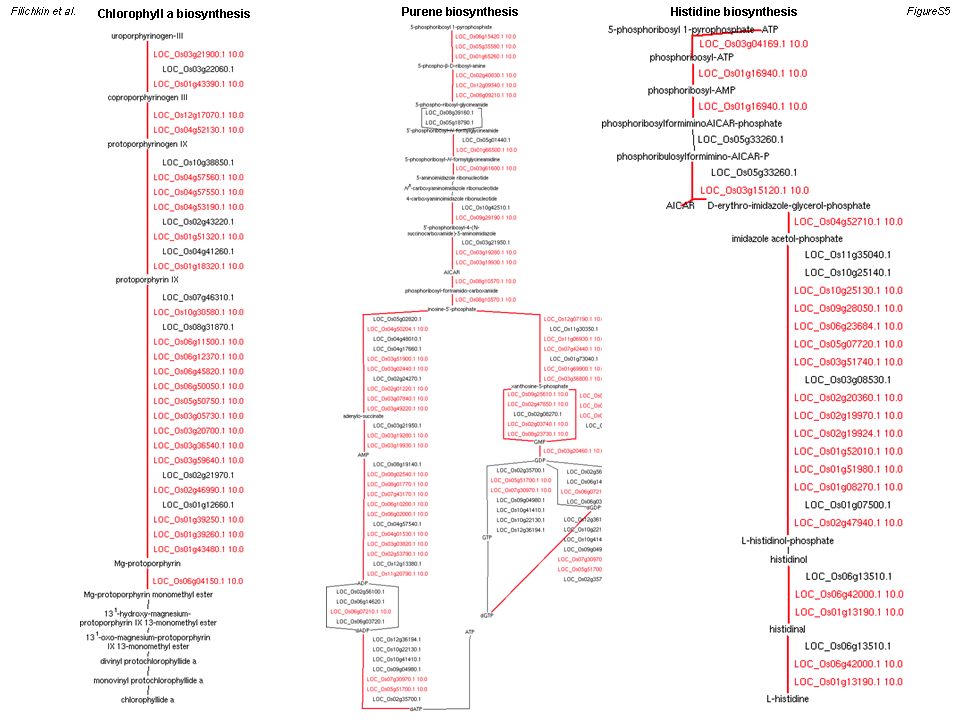

Supplement: Figure S5 — Expanded view of several pathways saturated with diurnally regulated genes. Cycling genes are depicted in red. (TIF) [file pone.0016907.s005.tif]

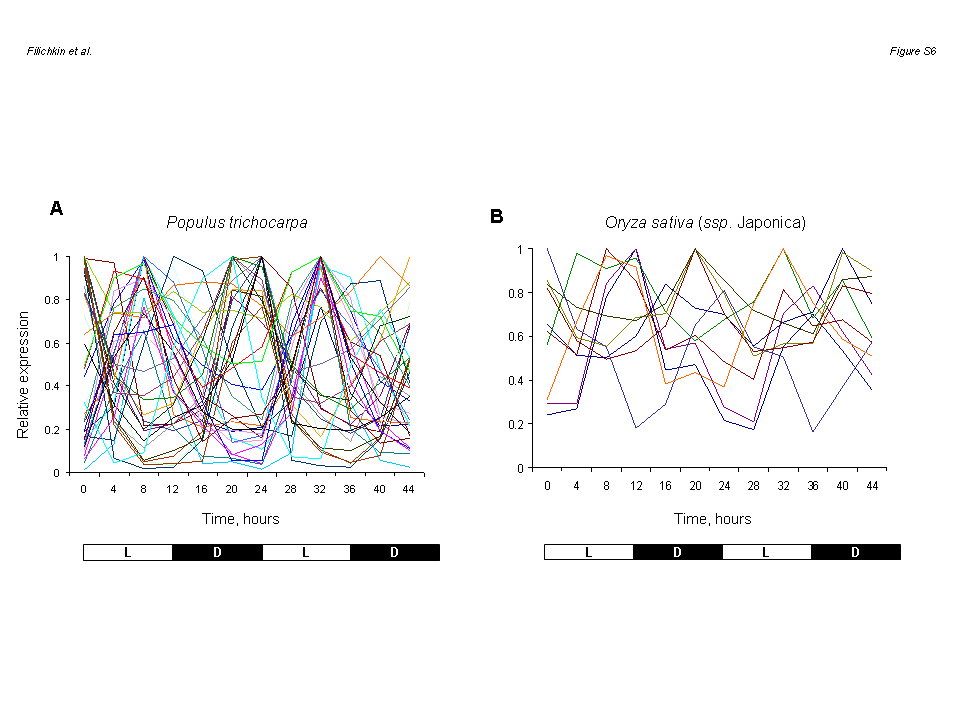

Supplement: Figure S6 — An example of the rhythmic expression among predicted poplar-rice TF orthologs. The 41 poplar TFs were arbitrarily selected based on high amplitude of oscillation and robust cycling under all tested diurnal conditions. Eleven out of 41 poplar TFs were identified as putative rice orthologs. The poplar TFs and corresponding rice orthologs show robust cycling profiles that encompass most of the phases of the day in the LDHH condition. Note clusters of TFs peaking at the light (L)/dark (D) transitions (dawn and dusk). Gene models corresponding to the predicted Populus trichocarpa and Oryza sativa (ssp. japonica) TFs were identified using the Databases of Poplar and Rice Transcription Factors (http://dptf.cbi.pku.edu.cn/). (TIF) [file pone.0016907.s006.tif]

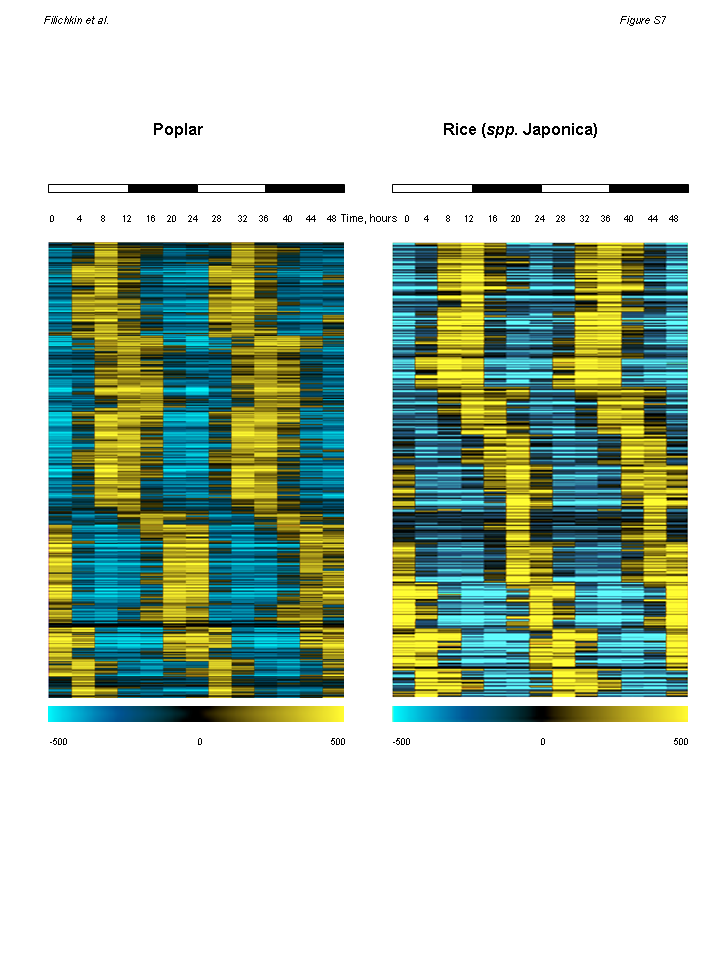

Supplement: Figure S7 — Peak expression of circadian-regulated genes occurs at all phases of the day. Expression heat maps of poplar (Populus trichocarpa) and rice (Oryza sativa, ssp. japonica) transcripts oscillating under photocyles (LDHH). Note even distribution of the clusters of peaking genes across all times of day. Mean centered expression levels are shown in yellow (high expression) and blue (low expression). The input gene list was generated using HAYSTACK with a Pearson correlation coefficient cutoff value r≥0.9. (TIF) [file pone.0016907.s007.tif]
