# Supplementary material for: Global Profiling of Rice and Poplar Transcriptomes Highlights Key Conserved Circadian-Controlled Pathways and cis-Regulatory Modules
Source: PLoS One. 2011 Jun 9;6(6):e16907. doi: 10.1371/journal.pone.0016907 (PMC3111414; doi:10.1371/journal.pone.0016907)
Supplement: Table S2 — Summary of Oryza sativa (ssp. japonica) GO category enrichment under photocyles (LDHH). (PDF) [file pone.0016907.s009.pdf]

Table S2. Summary of rice (spp. *Japonica)* GO category enrichment under photocyles (LDHH).

GO-ID	GO category	P-value	Correlation P-value	Cluster frequency	Overall frequency
166	nucleotide binding	1.17E-29	1.12E-27	709/3352 21.1%	4284/29753 14.3%
6519	amino acid and derivative metabolic process	4.20E-26	2.02E-24	140/3352 4.1%	490/29753 1.6%
5737	cytoplasm	3.87E-25	1.24E-23	501/3352 14.9%	2881/29753 9.6%
5623	cell	6.37E-22	1.53E-20	1446/3352 43.1%	10591/29753 35.5%
5575	cellular_component	1.61E-18	3.08E-17	1472/3352 43.9%	11008/29753 36.9%
5478	transporter activity	6.76E-15	1.08E-13	279/3352 8.3%	1581/29753 5.3%
6810	transport	2.51E-14	3.44E-13	403/3352 12.0%	2510/29753 8.4%
5622	intracellular	6.85E-12	8.21E-11	1000/3352 29.8%	7431/29753 24.9%
16301	kinase activity	2.82E-11	3.01E-10	327/3352 9.7%	2055/29753 6.9%
16020	membrane	2.25E-10	2.16E-09	580/3352 17.3%	4076/29753 13.6%
19725	cellular homeostasis	1.32E-09	1.15E-08	45/3352 1.3%	155/29753 0.5%
6464	protein modification process	1.76E-09	1.41E-08	315/3352 9.3%	2038/29753 6.8%
19748	secondary metabolic process	4.57E-07	3.37E-06	38/3352 1.1%	145/29753 0.4%
9056	catabolic process	2.02E-06	1.38E-05	122/3352 3.6%	716/29753 2.4%
6416	translation	2.48E-06	1.54E-05	151/3352 4.5%	931/29753 3.1%
5975	carbohydrate metabolic process	2.57E-06	1.54E-05	152/3352 4.5%	939/29753 3.1%
5739	mitochondrion	8.24E-06	4.65E-05	72/3352 2.1%	382/29753 1.2%
8150	biological_process	9.20E-05	4.90E-04	2566/3352 76.5%	21984/29753 73.8%
9536	plastid	2.63E-04	1.33E-03	93/3352 2.7%	578/29753 1.9%
5730	nucleolus	3.82E-04	1.84E-03	7/3352 0.2%	14/29753 0.0%
8135	translation factor activity	9.44E-04	4.31E-03	31/3352 0.9%	154/29753 0.5%
45182	translation regulator activity	1.05E-03	4.60E-03	31/3352 0.9%	155/29753 0.5%
6629	lipid metabolic process	1.27E-03	5.30E-03	105/3352 3.1%	698/29753 2.3%
19818	peroxisome	3.59E-03	1.43E-02	8/3352 0.2%	24/29753 0.0%
5783	endoplasmic reticulum	5.58E-03	2.14E-02	32/3352 0.9%	179/29753 0.6%
15979	photosynthesis	6.78E-03	2.50E-02	37/3352 1.1%	217/29753 0.7%
5840	ribosome	1.25E-02	4.45E-02	106/3352 3.1%	761/29753 2.5%
